# The Impact of Local Control on Overall Survival after Y-90 Selective Internal Radiotherapy of Liver Metastases in Oligometastatic Cancer: A Retrospective Analysis

**DOI:** 10.3390/cancers16132401

**Published:** 2024-06-29

**Authors:** John Yeakel, Steven N. Seyedin, Garrett Harada, Garo Hagopian, Sharmeen Mahmood, Rebecca Bennett, Jeremy P. Harris, Elliot M. Abbott, Sydney Lindner, Farshid Dayyani, Varun Sehgal, Jeffrey V. Kuo, Nadine Abi-Jaoudeh

**Affiliations:** 1 Department of Radiation Oncology, University of California Irvine, Orange, CA 92868, USA; yeakel.john@mayo.edu (J.Y.);; 2 Department of Radiation Oncology, Mayo Clinic, Rochester, MN 55902, USA; 3 Department of Medicine, University of California Irvine, Orange, CA 92868, USA; 4 Division of Hematology/Oncology, Department of Medicine, University of California Irvine, Orange, CA 92868, USA; 5 Division of Vascular and Interventional Radiology, Department of Radiological Sciences, University of California Irvine, Orange, CA 92868, USA; rzbennet@hs.uci.edu (R.B.);; 6 MIM Software Inc., Cleveland, OH 44122, USAslindner@mimsoftware.com (S.L.)

**Keywords:** oligometastatic, local control, SIRT, liver metastases, OS, Y-90

## Abstract

**Simple Summary:**

Y-90 Selective Internal Radiotherapy (SIRT) uses injectable radioactive microspheres to treat inoperable liver metastases. It is poorly understood how the effective local control of small-volume metastatic disease with Y-90 SIRT may improve patient survival. Here, we demonstrate that failure to locally control liver metastases with ytrritum-90 (Y-90) SIRT is associated with worse 1-year overall survival for patients with ≤5 metastases, emphasizing the importance of disease control in those with early metastatic disease.

**Abstract:**

Y-90 Selective Internal Radiotherapy (SIRT) is an ablative therapy used for inoperable liver metastasis. The purpose of this investigation was to examine the impact of local control after SIRT on overall survival (OS) in oligometastatic patients. A retrospective, single-institution study identified oligometastatic patients with ≤5 non-intracranial metastases receiving unilateral or bilateral lobar Y-90 SIRT from 2009 to 2021. The primary endpoint was OS defined from Y-90 SIRT completion to the date of death or last follow-up. Local failure was classified as a progressive disease at the target lesion(s) by RECIST v1.1 criteria starting at 3 months after SIRT. With a median follow-up of 15.7 months, 33 patients were identified who had a total of 79 oligometastatic lesions treated with SIRT, with the majority histology of colorectal adenocarcinoma (*n* = 22). In total, 94% of patients completed the Y-90 lobectomy. Of the 79 individual lesions treated, 22 (27.8%) failed. Thirteen patients received salvage liver-directed therapy following intrahepatic failure; ten received repeat SIRT. Median OS (mOS) was 20.1 months, and 12-month OS was 68.2%. Intralesional failure was associated with worse 1 y OS (52.3% vs. 86.2%, *p* = 0.004). These results suggest that intralesional failure following Y-90 may be associated with inferior OS, emphasizing the importance of disease control in low-metastatic-burden patients.

## 1. Introduction

Approximately 122,000 patients per year are diagnosed with metastatic cancer with liver involvement with a 1-year survival of approximately 15% [1]. Most liver metastases are either inoperable at the time of presentation or the patients are medically unfit for surgery [2]. Employing ablative locoregional therapy for liver metastasis in patients with low disease burden can improve overall survival [3]. Multiple liver-directed therapies are available, including trans-arterial radioembolization (TARE), also known as selective internal radiotherapy (SIRT), using yttrium-90 (Y-90) microspheres [4]. Y-90 TARE has been reported to achieve local control rates of approximately 75% [5,6].

The prognosis of patients with liver metastases correlates with the burden of disease in the liver, with the longest survival rates in patients with only one to three liver metastases [7]. Recent studies exploring outcomes in oligometastatic disease note that local control of the metastases is key to improving survival outcomes in these patients [6,8]. However, the impact of hepatic lesional control (LC) data on overall survival (OS) after SIRT in the oligometastatic remains unknown. We hypothesized that LC after Y-90 correlates with improved OS in oligometastatic patients with unresectable liver metastases. Additionally, we examined our institutional Y-90 TARE experience to identify factors that affect LC and determine its effect on OS.

## 2. Materials and Methods

This was a retrospective, single-institution, IRB-approved study (2021–6535) that included synchronous (liver metastases at diagnosis) and metachronous (liver metastases development after primary tumor treatment) metastatic patients with limited disease burden, defined as 5 or fewer metastases, who underwent Y-90 TARE from 2009 to 2021 with resin or glass microspheres. These patients received Y-90 therapy at the recommendation of our institutional hepatobiliary tumor criteria. Patients with intra-cranial metastases before Y-90 therapy were excluded. Since this is a quaternary referral center, patients who did not return for imaging follow-up or who had less than a 3-month follow-up at our center were excluded. The primary endpoint was OS defined from Y-90 TARE completion to the date of death or last follow-up. Treatment toxicity was considered outside the scope of this paper and is not reported. However, none of the patients in this study experienced grade 3+ or higher radioembolization-induced liver toxicity.

Patients were treated with resin or glass Y-90 microspheres according to previously published methodologies [9,10,11]. Specifically, all cases were discussed at a multidisciplinary tumor board consisting of radiation oncologists, interventional radiologists, diagnostic radiologists, hepatobiliary surgeons, medical oncologists, and clinical research personnel. Cases deemed appropriate were then scheduled for an initial hepatic angiogram to determine tumor vascular supply, identify extra-hepatic arteries emanating from the tumoral vascular supply that may cause iatrogenic gastro-intestinal radiation ulcers if not embolized, and determine the volume of tumor and liver to be treated. Further, a tracer dose of Tc-99m macro-aggregate albumin (MAA) was injected into the proposed treatment vessel(s) followed by SPECT to determine the lung-shunting fraction.

The method by which the Y-90 activity was calculated differed according to the microsphere type (resin or glass) and treatment type (lobar or segmental) and evolved during the study period. For resin microspheres, the Body Surface Area (BSA) method was almost exclusively utilized. The activity to be administered to the target lobe was based on:

(1) $$Prescribed\;Activity\left(GBq\right)=\left(BSA-0.2\right)+\left[\left(\frac{tumor\;mass}{total\;liver\;mass}\right)\times 100\right]$$

For patients who have received ≥2 courses of multi-agent chemotherapy, the BSA-prescribed activity was reduced empirically by up to 30% to reduce the risk of radiation-induced liver disease.

For glass microspheres, the medical internal radiation dose (MIRD) model was used:

(2) $$Prescribed\;Activity\left(GBq\right)=\frac{Target\;Dose\left(Gy\right)\times liver\;mass\left(kg\right)}{\left[50\times \left(1-Lung\;Shunting\;Fraction\right)\times \left.(1-percent\;residual\;after\;infusion\right)\right]}$$

where the liver mass was determined from the pre-treatment CT or MRI. The lung-shunting fraction was determined as described above, and the percent of residual activity after infusion was estimated based on similar prior treatments. No adjustments were made for prior cytotoxic chemotherapy. For segmental treatments, the activity was derived from calculating the dose for the entire lobe, even though it was given selectively to a single segment [12,13,14]. The prescribed radiation dose was defined using guidelines provided by the European Association of Nuclear Medicine with a goal of 80–150 Gy for radiation lobectomy and up to 400 Gy for segmentectomy [15].

Liver lesions were included in the analysis if noted by the reading radiologist, including if they were measured to be less than 1 cm. While not technically included in RECIST criteria, they were designated as metastatic lesions and were included in the decision for local treatment. Because of this, their response to treatment was followed. Lesional failure (LF) was defined as progressive disease at treated target lesion(s) by RECIST v1.1 criteria starting at three months from the date of the last Y-90 procedure in case of multiple treatments (bilobar or multiple segments). To account for possible pseudo-progression on imaging due to treatment-induced necrosis and/or edema, the lesion diameter has to have increased on at least 2 consecutive imaging studies. The three-month post-treatment timeframe was established due to the mean time to pseudo-progression being found to be 30 days and the possibility for these changes to last for several months [16]. Patients who did not demonstrate failure after Y-90 TARE were classified as achieving LC. Treated lesions receiving salvage locoregional therapy were also considered to have failed. Patients’ clinical outcomes were calculated only from the time of the first treatment; no outcome data regarding the re-treatment of lesions were included in our study.

Pre- and post-treatment CT, MRI, and PET/CT imaging were imported into the MIM contouring software system (MIM Software Inc., Cleveland, OH). Tumor and liver volumes were then delineated by a radiation oncologist (S.N.S. or J.Y.). For those with pre-treatment Tc-99 macroaggregated albumin (MAA) SPECT/CT, lesional dosimetry was evaluated with 3D voxel-based dose estimation using the MIM SurePlan^TM^ suite. Three-dimensional voxel-based dosimetry was performed using the local deposition method (LDM) with normalization of the dose region based on the known injected Y-90 activity and the total SPECT counts in the delineated liver and lungs. Post-treatment Y-90 SPECT or PET imaging was not available at the start of the study timeline. The voxelized biological effective dose (BED) conversion and the dose received by 100% of the tumor volume (D100%) were calculated for each lesion. BED conversion was performed using the linear-quadratic (LQ) model designed for low-dose-rate (LDR) brachytherapy (Table S1).

Statistical analysis was performed using Stata version 18.0 (StataCorp, College Station, TX, USA). OS and LC outcomes were calculated using the Kaplan–Meier method, and univariate analysis was conducted via log-rank testing. For OS, patients were censored by date of death or last follow-up, and for LC, patients were censored by date of failure, death, or last follow-up. Univariate analysis of outcomes by patient characteristic variables was also conducted using Kaplan–Meier and log-rank testing. Non-parametric Mann–Whitney testing was performed to compare median values between patients with and without local failure after Y-90. Univariate regression was performed to assess the effect of D100% on LC for cases with lesional failure. The threshold of significance was set at *p* < 0.05.

## 3. Results

With a median follow-up of 15.7 months, a total of 33 patients with 79 liver lesions were eligible for inclusion in this study. Upon volumetric analysis of the liver tumor burden, the range of liver involvement was 0.03% to 56.27%. The median lesion volume was 2.72 cm^3^ (range: 0.19–1024.21 cm^3^). Patient characteristics are further described in Table 1.

### 3.1. Local Control

**Figure 1:**
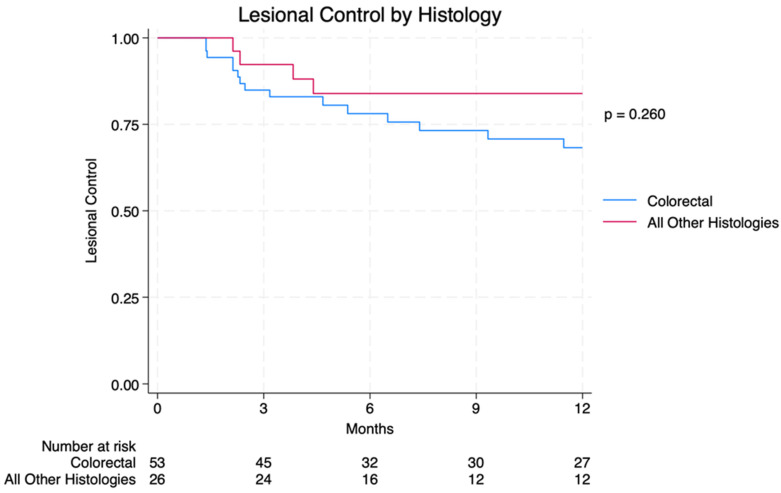
Lesional control by histology.

Of the 79 individual lesions treated, 22 (27.8%) failed. The twelve-month LC rate was 76.0% and the 18-month LC rate was 73.4%. Thirteen patients received salvage liver-directed therapy following intrahepatic failure, of which 10 received repeat Y-90 TARE. Upon further examination by histologic subtype, 12-month LC for colorectal metastases (CRC, *n* = 53 lesions) was 71.7% compared to 84.6% for other histologies (*p* = 0.207) (Figure 1). For CRC patients, 12-month LC rates for right- vs. left-sided primary lesions were 61.5% vs. 75.0% (*p* = 0.349); the median duration of LC (mLC) was 6.5 months vs. not reached for right- and left-sided primaries, respectively (Figure S1). The twelve-month LC for right vs. left hepatic lobe lesions was 82.4% vs. 64.3% (*p* = 0.072); mLC was not reached for right-lobe lesions while mLC was 25.8 months for left-lobe lesions (Figure S2). The median volume of treated lesions that failed was significantly higher for controlled metastases (17.2 cc vs. 1.99 cc, *p* < 0.001). Metastases smaller than 10 cc were more likely to exhibit LC compared to those 10 cc or larger (83.6% vs. 33.3%, *p* < 0.001). Additional LC data are listed in Table 2.

Tm-99 MAA records were available for analysis in 18 patients with 43 treated lesions, of which 9 patients with 10 lesions failed the following treatment. The median activity delivered was 1.42 GBq (Range = 0.46–3.12). The median lesion D100% was 20.0 Gy. The median D100% was 9.6 vs. 20.5 Gy for treated metastases with LF vs. without. The difference between the median D100% of these groups was not significant (*p* = 0.233). Linear regression analysis of the duration of LC vs. D100% for lesions that failed following TARE showed a weak positive correlation (ß = 0.17; 95% CI = 0.02–0.36; *p* = 0.079; R^2^ = 0.337).

### 3.2. Overall Survival:

**Figure 2:**
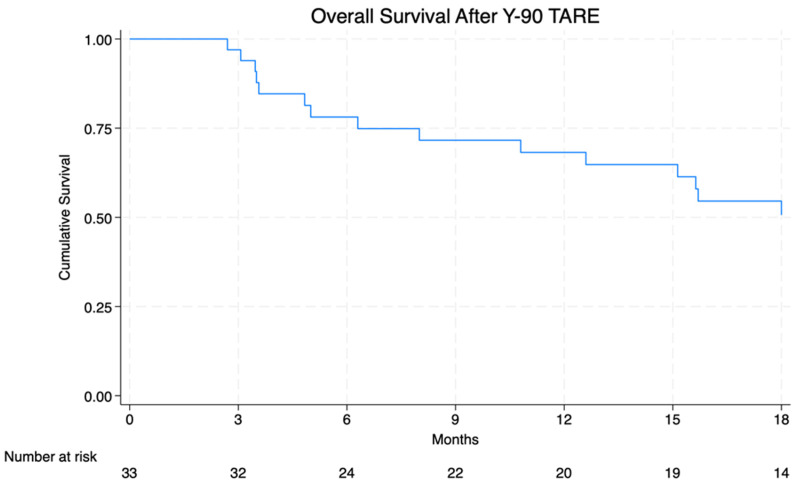
Overall survival after Y-90 TARE (trans-arterial radioembolization).

**Figure 3:**
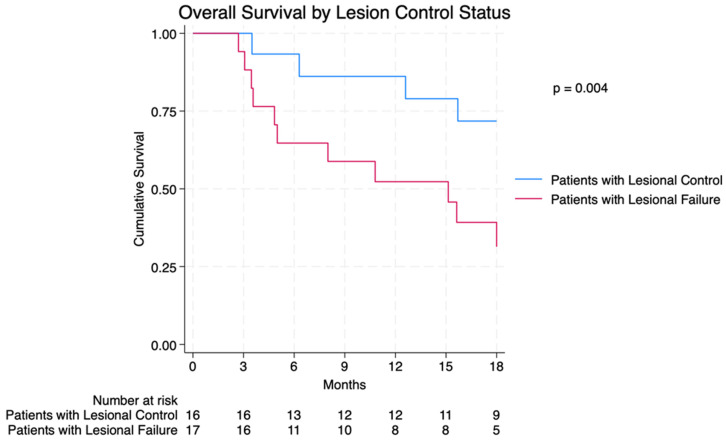
Overall survival by lesion control status.

Median OS (mOS) was 20.1 months (95% CI: 10.8–32.3 months), 12-month OS was 69.7%, and 18-month OS was 54.6% (Figure 2). Patients with LF after TARE were found to have worse mOS (15.1 months vs. 75.5 months) and 12-month OS (52.9% vs. 87.5%) (*p* = 0.004) (Figure 3). In CRC patients, those with CEA greater than the median of 6.4 ng/mL had a 12-month survival of 64.3% vs. 100.0% for those with CEA <6.4 ng/mL (*p* = 0.054) (Figure S3). Eleven patients did not receive post-SIRT chemotherapy, and 5 of these patients experienced intralesional failure. There was no significant difference between OS and LC for patients who received post-Y-90 chemotherapy and those who did not (mOS 20.1 vs. 15.7 months, *p* = 0.598) (LC 54.6% vs. 45.5%, *p* = 0.622). Additionally, the mOS of those with and without extrahepatic disease was not significantly different (15.1 mo vs. 20.1, *p* > 0.5).

## 4. Discussion

This retrospective investigation in oligometastatic patients demonstrated that LF after TARE was correlated with inferior OS. Patients with control metastases were found to have a 12-month OS of 87.5% compared to their relapsed counterparts with an mOS of 75.5 months. This finding was expected, as metastatic-directed therapy has been shown to improve OS in those with limited disease burden [17]. Multiple studies have examined prognostic factors for OS after liver TARE for CRC metastasis. Focused exclusively on those with oligometastatic disease, this study demonstrated a correlation between LC and OS.

Sankhla et al. examined a cohort of 45 patients treated with Y-90 TARE for CRC metastases and observed that an objective response at 3 months by RECIST criteria was prognostic for OS [18]. However, 71% of this cohort of patients had >10 liver metastases at the time of treatment. In the current study, LF at any timepoint was correlated with OS, including in patients with an initial response at the 3-month timepoint noted by Sankhla et al. Failure after that time window was still a contributing factor to shortened OS.

Examining a similar oligometastatic cohort, Kurilova et al. noted 1-year OS was 90% while LF was only 20%, superior to the nearly 28% LF rate in this study [6]. While this study included up to five metastases per patient, their group only allowed a maximum of three liver metastases. Our study noted lower LC correlated with worse OS, implying that superior LC improves OS. Kurilova et al., however, did not examine the link between OS and LF, likely due to the limited sample size. Padia et al. noted a similar LC rate of 71% in a cohort of patients with a similar disease burden, although not strictly oligometastatic (median number of liver metastases of 1.5 and 95% of patients had <20% liver involvement) [5]. The OS at 1 year was 80%, superior to the OS rate in this study. The superior OS in Padia’s group could be attributed to a lower median tumor burden compared to this study and the large proportion of neuroendocrine tumor patients (*n* = 10, 28%) who have a longer overall survival in general [19,20]. Additionally, 94% of patients in this study underwent radiation lobectomy with lower doses compared to radiation segmentectomy, while Padia’s and Kurilova’s were segmentectomies with higher doses, reinforcing the importance of dosimetry [15].

Data examining OS after first-line chemotherapy and SIRT for limited-volume metastatic colorectal cancer can be found in three large, international, randomized trials, namely FOXFIRE, SIRFLOX, and FOXFIRE-GLOBAL. These studies randomized patients to receive chemotherapy alone vs. chemotherapy and SIRT to the liver with the primary endpoint of OS analyzed in the intention-to-treat grouping. A combined analysis of the results of these three trials examined 549 patients who received FOLFOX alone vs. 554 patients who received FOLFOX plus SIRT. With a median follow-up of 43.3 months, the OS between groups was not statistically significant (mOS 23.3 vs. 22.6 months in the FOLFOX and FOLFOX plus SIRT groups, respectively (hazard ratio of 1.04, 95% confidence interval of 0.90–1.19, *p* = 0.61)). Examination of this clinical cohort shows two-thirds of patients had liver-only metastatic disease burden and <25% of total liver volume involved in metastases. As noted by the authors, selective use of SIRT as first-line treatment of unresectable colorectal liver metastases is necessary, as they did not find a difference in OS between groups [21]. The patient cohort in our study had a median liver involvement of 0.92%, as determined by the volumetric measurement of liver and metastases on pre-treatment CT, and liver lesions involving three or more segments or measuring >100 cm^3^ had poor LC rates (Table 2). It may be that despite liver-only involvement in the majority of the included patients, the liver volume involved by the disease was still too high, even when the majority of patients had <25% of liver involved.

Among various treatment and patient characteristics examined in this study, LC was found to correlate with OS. An earlier study published by Kurilova et al., examining prognostic factors affecting outcomes of Y-90 TARE in heavily pre-treated patients with CRC metastases, found that CEA, albumin, ALT, the degree of tumor differentiation, and the sum of the two largest tumor diameters were independently associated with OS in a multivariate analysis [22]. Other studies have supported these findings, publishing evidence that AST/ALT, CEA, and performance status are important prognostic factors for patients undergoing Y-90 TARE [23,24]. Due to the small sample size and inclusion of multiple histologies, an analysis of these factors was not performed. Previously, studies did not attempt to prognosticate OS after Y-90 TARE due to small sample sizes [5,6].

Because LC was associated with superior OS in this study, understanding the risk factors of failure is important. Our results suggest a possible correlation between the size and location of the lesion and LC, as lesions with a volume of ≥10 cm^3^ had lower LC rates. Schonewolf et al. found a correlation between tumor volume <300 mL and a decreased pattern of intrahepatic failure following Y-90 TARE (*p* = 0.046) [25]. Kurilova et al. reported that longer liver progression-free survival was associated with improved OS [22]. Gulec et al. showed double the length of survival in patients with a total tumor volume of <200 cm^3^ compared with those with >200 cm^3^ (*p* < 0.05) [26]. Other studies have correlated a combined diameter of ≥10 cm for a patient’s two largest hepatic lesions treated with Y-90 with a higher probability of mortality within 12 months of Y-90 [22,27]. The extent of liver involvement with the tumor has been repeatedly shown to have an inverse association with OS, but the effect of LC on OS was not reported [22,28,29,30]. One explanation for the unexplored LC correlation in these patients is due to impaired liver function impacting OS alone from the significantly higher disease burden compared to the patients from this retrospective study.

In our study, the minimum global tumor dose (D100%) was not found to be statistically significant between lesions that failed vs. those that achieved durable control, but there was a weakly positive correlation between D100% and time to failure (R^2^ = 0.33). This minimum global tumor dose represents the homogeneity of the deposited dose to the treated lesions as predicted by the Tc-99 MAA SPECT/CT pre-treatment imaging. The predictive accuracy of pre-treatment Tc-99 MAA SPECT/CT imaging is controversial, with differences in pre- and post-dose distributions being attributed to changes in local tumor vasculature between pre- and post-treatment scans, different intravascular kinetics of the microspheres vs. Tc-99 MAA particles, and changes in the position of the catheter during the injection of Tc-99 MAA vs. Y-90 microspheres [31]. Dewaraja and colleagues generated a tumor control probability model from 89 HCC and metastases treated with Y-90 utilizing post-treatment PET/CT voxel-based dosimetry and noted that mean tumor doses above 292 Gy corresponded to greater than 50% local control [32]. This model has been integrated into a clinical trial for external beam dose escalation of HCC and could be similarly considered for underdose liver metastases as well (NCT04518748) [33].

Because the dose distribution of Y-90 is dependent on the arterial flow, non-uniform perfusion of tumors could lead to local failure in areas not treated with a sufficient dose. However, the importance of dose uniformity represented by the D100% on voxel-based post-treatment dosimetry imaging has not been fully investigated. Sanhkla et al. examined post-treatment dosimetry with Y-90 SPECT/CT and noted similar sensitivity and specificity of the mean tumor dose (TD), max TD, and minimum TD after an analysis of the treatment response via receiver operating characteristic analysis [18]. In a report by van den Hoven et al., changes in total lesion glycolysis (TLG) on FDG-PET CT before and after Y-90 TARE showed that a minimum TD of 40–60 Gy was required to achieve a 50% reduction in TLG [34]. Further examination of the dose–response relationship curve found minimal metabolic activity after a mean TD of 100 Gy, which was then used as a basis of the target dose recommended in recently released guidelines for resin microsphere-based Y-90 therapy [15]. Allimant et al. studied tumor dosimetry with treatment planning systems designed for Y-90 TARE for hepatocellular carcinoma and showed that incomplete tumor coverage contributed to poor LC, which was associated with decreased OS and PFS [35]. Our results mirror these findings, with the LF of treated metastatic tumors being associated with decreased OS within our cohort.

Our study has several inherent limitations, including the retrospective design and analysis of only a single institution. While excluding patients with less than 3 months of post-treatment follow-up could be a source of potential bias affecting OS results due to death, the low tumor burden and favorable performance status of the patients in this cohort would make this very rare. Those with extrahepatic disease presented with a lower mOS but it was not meaningfully different compared to those with a liver-confined disease. However, the primary benefit of Y-90 for those with oligometastatic intrahepatic disease alone could be obscured due to the small sample size. Our dosimetry was evaluated using only pre-treatment MAA SPECT imaging, without the use of post-treatment imaging, which is more informative about the actual dose deposition. Our patients were also initially treated using the BSA method for activity prescription, which has inherent inaccuracies, as discussed. By using RECIST 1.1 criteria to evaluate the tumor response, target lesions are supposed to be >1 cm in diameter, but many of the tumors were smaller than this size cutoff, which could reduce the accuracy of the objective response. Challenges in assessing post-treatment imaging included labeling pseudo-progression as LF [36]. In this study, the evaluation of LF lesions took place a minimum of 3 months post-treatment or at the time of symptomatic progression requiring intervention to avoid such misclassification errors.

## 5. Conclusions

In conclusion, this study demonstrated that LF following Y-90 TARE could be associated with inferior OS in patients with oligometastatic disease. Large tumor volumes are at increased risk of local failure, but prospective studies with larger populations are needed for validation.

## Figures and Tables

**Table 1:** Patient characteristics.

	N/(Median)	%/(Range)		N/(Median)	%/(Range)
Number of Patients	33	100.0	Metastasis-Directed Therapy (Prior To Y-90)		
Number of Metastases	79	100.0	Partial Hepatectomy	9	45.0
Tumors per Patient	(3.0)	(1.0–5.0)	TACE	2	10.0
Tumor Volume (cm^3^)	(2.72)	(0.07–2046.82)	Ablation ^b^	9	45.0
Liver Involvement (%)	(0.92)	(0.03–56.27)	Systemic Therapy (Prior to Y-90)		
Follow up (months)	(15.7)	(2.7–75.5)	Single Agent Chemotherapy	1	3.2
Sex			Multi-Agent Chemotherapy	13	41.9
Men	22	66.7	Targeted Agent Monotherapy	4	12.9
Women	11	33.3	Immunotherapy	1	3.2
Age (years)	(62.1)	(37.7–86.6)	Targeted Agent and/or Immunotherapy + Chemotherapy	12	38.7
Karnofsky Performance Status			Number of Previous lines of Systemic Therapy		
100	8	24.2	0	1	3.7
90	12	36.4	1	16	59.3
80	11	33.3	2	6	22.2
70	2	6.1	3	3	11.1
Primary Malignancy			4	0	0.0
Colorectal	22	66.7	5	1	3.7
Pancreatic	7	21.2	Y-90 Preparation		
Neuroendocrine	1	3.0	Glass Microspheres	3	9.1
Uveal Melanoma	1	3.0	Resin Microspheres	30	90.9
Thymic Carcinoma	1	3.0	Y-90 TARE Procedure		
Gastric Adenocarcinoma	1	3.0	Bilateral Lobectomy	24	72.7
Timing of Metastasis			Unilateral Lobectomy	7	21.2
Synchronous	15	45.5	Segmentectomy	2	6.1
Metachronous	18	54.5	Patterns of Failure (After First Y-90 Treatment)		
Distribution of Metastases			Lesional Failure	7	21.2
Extra + Intrahepatic Disease	6	18.2	New hepatic Lesion(s)	14	42.4
Intrahepatic Disease Only	27	81.8	New Extrahepatic Lesion(s)	5	15.2
Liver Markers (Prior To Y-90)			Lesional Failure and New Hepatic Lesion(s)	3	9.1
AST (U/L)	25.5	(12.0–65.0)	New Hepatic and Extrahepatic Lesion(s)	0	0.0
ALT (U/L)	23.5	(6.0–228.0)	Lesional Failure and New Hepatic and Extrahepatic Lesion(s)	1	3.0
Albumin (g/dL)	4.1	(2.9–4.9)	None	3	9.1
CEA (ng/mL) ^a^	6.4	(0.8–618.8)	Patients Who Received Repeat Y-90	10	30.3

AST = Aspartate aminotransferase; ALT = Alanine transaminase; CEA = Carcinoembryonic antigen; Y-90 = Yttrium-90; TACE = Transarterial Chemoembolization; TARE = Transarterial Radioembolization. ^a^ CEA laboratory values apply only to patients with metastatic colorectal cancer primary disease; ^b^ Ablation includes radiofrequency ablation, cryoablation, microwave, alcohol, and thermal techniques.

**Table 2:** Local Control Outcome Characteristics.

	N/(Median)	%/(Range)	*p*-Value
Lesion Volume by Control Status			
Lesional Failure (N = 22) (cm^3^)	(17.21)	(0.46–246.82)	**<0.001**
Lesional Control (N = 57) (cm^3^)	(1.99)	(0.07–156.03)	
LC Rate by Lesion Volume			
≥10 cm^3^	18	33.3	**<0.001**
<10 cm^3^	61	83.6	
≥100 cm^3^	8	12.5	**<0.001**
<100 cm^3^	71	78.9	
LC Rate by Involved Liver Segment			
Segment 1	4	75.0	**<0.001**
Segment 2	7	100.0	
Segment 3	2	0.0	
Segment 4a	7	42.9	
Segment 4b	3	33.3	
Segment 5	7	85.7	
Segment 6	9	88.9	
Segment 7	14	100.0	
Segment 8	8	75.9	
LC Rate Stratified by Number of Segments Involved			
2 segments	13	69.0	0.340
1 segment	61	78.7	
≥3 Segments	5	0.0	**<0.001**
<3 Segments	74	77.0	
Duration of LC by Receipt of Post-Y-90 Chemotherapy (months)			
Received Chemotherapy (N = 22)	(20.1)	(3.5–75.5)	0.890
No Chemotherapy (N = 11)	(16.2)	(2.7–54.3)	

Univariate Mann-Whitney U and log-rank assessments of lesion volume, segment involvement, and receipt of post-y-90 chemo-therapy on outcomes of local control are displayed with bolded values indicating statistical significance at *p* < 0.05. LC = Local Control; Y-90 = Yttrium-90.

## Data Availability

The data presented in this study are available upon request from the corresponding author.

## Supplementary Materials

The following supporting information can be downloaded at: https://www.mdpi.com/article/10.3390/cancers16132401/s1, Figure S1: Lesional control rate by laterality of colorectal primary tumor, Figure S2: Lesional control by hepatic lobe. Figure S3: Overall survival by CEA level. Table S1: Biologic Effective Dose Calculation Model.
