# Supplementary material for: The Impact of Local Control on Overall Survival after Y-90 Selective Internal Radiotherapy of Liver Metastases in Oligometastatic Cancer: A Retrospective Analysis
Source: Cancers (Basel). 2024 Jun 29;16(13):2401. doi: 10.3390/cancers16132401 (PMC11240767; doi:10.3390/cancers16132401)
Supplement: Supplementary file 1 [file cancers-16-02401-s001.zip › cancers-3062658-supplementary.pdf]

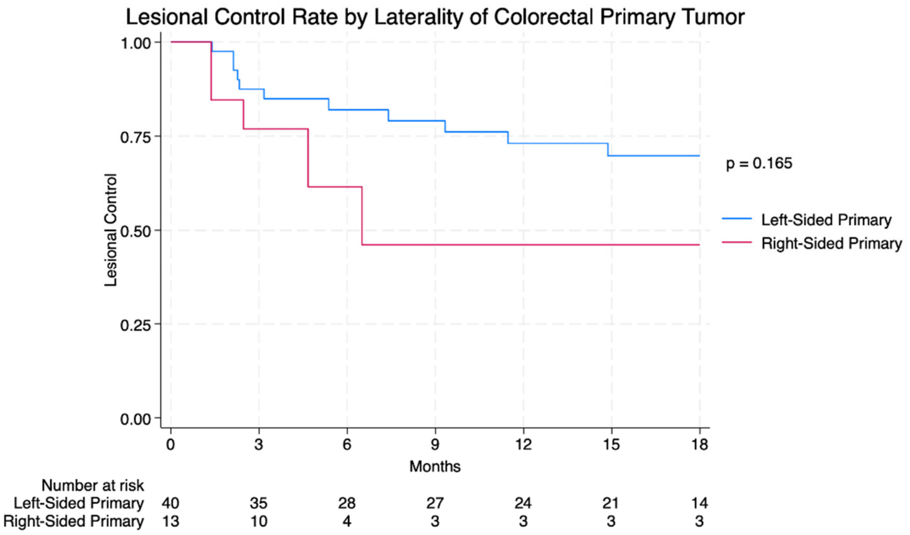

Figure S1. Lesional Control Rate by Laterality of Colorectal Primary Tumor.

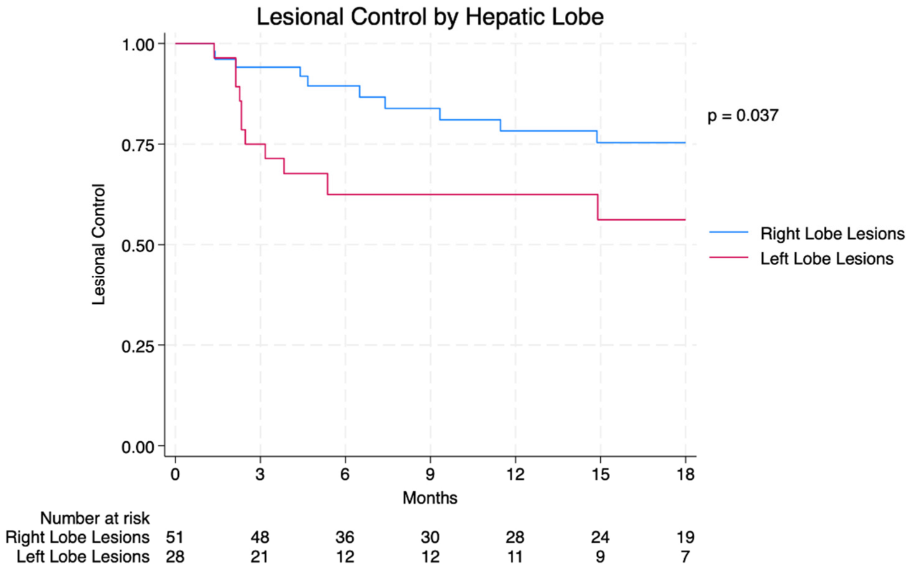

Figure S2. Lesional Control by Hepatic Lobe

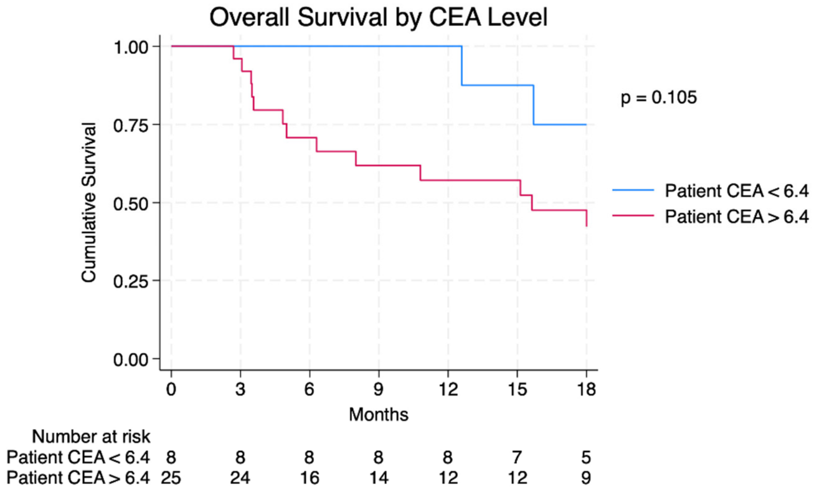

Figure S3. Overall Survival by CEA Level.

Table S1. Biologic Effective Dose Calculation Model.

$BED = D\left[1 + 2\left(\frac{\frac{d_0 \times \lambda}{\alpha}}{\beta}\right)\left(\frac{k}{\mu - \lambda}\right)\right] - \left(0.693 \times \frac{T}{\alpha \times T_p}\right)$
$k = \left(\frac{1}{1 - \varepsilon}\right)\left[\left(\frac{1 - \varepsilon^2}{2\lambda}\right) - \left(\frac{1 - \varepsilon e^{-\lambda \times T_{eff}}}{\mu + \lambda}\right)\right]$
$T_{eff} = \frac{1}{\lambda} \times \ln\left(1.44 d_0 \alpha T_p\right)$
